# Biochemical and inflammatory biomarkers in ischemic stroke: translational study between humans and two experimental rat models

**DOI:** 10.1186/s12967-014-0220-3

**Published:** 2014-08-03

**Authors:** Patricia Martínez-Sánchez, María Gutiérrez-Fernández, Blanca Fuentes, Jaime Masjuán, María Alonso de Leciñana Cases, Maria Elena Novillo-López, Exuperio Díez-Tejedor

**Affiliations:** 1 Department of Neurology and Stroke Center, Neuroscience and Cerebrovascular Research Laboratory, La Paz University Hospital, Autonoma of Madrid University, Neurosciences Area of IdiPAZ Health Research Institute, Alcalá de Henares University, Madrid, Spain; 2 Department of Neurology, Stroke Unit, Ramón y Cajal Hospital, IRYCIS Health Research Institute, Madrid, Spain

**Keywords:** Brain ischemia, Chemokines, Animal models, Acute stroke, Cell death, Inflammation

## Abstract

**Background:**

our objective was to examine the plasma levels of three biological markers involved in cerebral ischemia (IL-6, glutamate and TNF-alpha) in stroke patients and compare them with two different rat models of focal ischemia (embolic stroke model- ES and permanent middle cerebral artery occlusion ligation model-pMCAO) to evaluate which model is most similar to humans. Secondary objectives: 1) to analyze the relationship of these biological markers with the severity, volume and outcome of the brain infarction in humans and the two stroke models; and 2) to study whether the three biomarkers are also increased in response to damage in organs other than the central nervous system, both in humans and in rats.

**Methods:**

Multi-center, prospective, case-control study including acute stroke patients (n = 58) and controls (n = 19) with acute non-neurological diseases Main variables: plasma biomarker levels on admission and at 72 h; stroke severity (NIHSS scale) and clinical severity (APACHE II scale); stroke volume; functional status at 3 months (modified Rankin Scale [mRS] and Barthel index [BI]). Experimental groups: ES (n = 10), pMCAO (n = 6) and controls (tissue stress by leg compression) (n = 6). Main variables: plasma biomarker levels at 3 and 72 h; volume of ischemic lesion (H&E) and cell death (TUNEL).

**Results:**

in stroke patients, IL-6 correlated significantly with clinical severity (APACHE II scale), stroke severity (NIHSS scale), infarct volume (cm^3^) and clinical outcome (mRS) (r = 0.326, 0.497, 0.290 and 0.444 respectively; *P* < 0.05). Glutamate correlated with stroke severity, but not with outcome, and TNF-alpha levels with infarct volume. In animals, The ES model showed larger infarct volumes (median 58.6% vs. 29%, *P* < 0.001) and higher inflammatory biomarkers levels than pMCAO, except for serum glutamate levels which were higher in pMCAO. The ES showed correlations between the biomarkers and cell death (r = 0.928 for IL-6; P < 0.001; r = 0.765 for TNF-alpha, P < 0.1; r = 0.783 for Glutamate, *P* < 0.1) and infarct volume (r = 0.943 for IL-6, *P* < 0.0001) more similar to humans than pMCAO. IL-6, glutamate and TNF-α levels were not higher in cerebral ischemia than in controls.

**Conclusions:**

Both models, ES and pMCAO, show differences that should be considered when conducting translational studies. IL-6, Glutamate and TNF-α are not specific for cerebral ischemia either in humans or in rats.

## Introduction

Experimental focal cerebral ischemia models have been developed in rats to mimic human stroke and serve as an indispensable tool in the stroke research field [1],[2]. However, although rats are ideal animals for mimicking human stroke due to the close similarities of their cerebrovascular anatomy and physiology with humans [3]–[5], numerous drugs have demonstrated efficacy in preclinical assessments but not in clinical trials in humans [2]. This failure of translation could be explained, at least in part, by differences in the ischemic response between animals and humans.

Ischemic injury trigger inflammatory cascades and changes in the neurotransmitters in the brain parenchyma that may further amplify the tissue damage. Interleukin-6 (IL-6) and tumor necrosis factor-alpha (TNF-α) are some of the most studied cytokines in stroke-related inflammation [6]–[10]. In stroke patients, IL-6 has been linked to early neurological deterioration (END) [6], greater infarct volumes [7] and poorer long-term outcome [8]. High levels of TNF-α in plasma also correlate also with infarct volume and neurological function in models of cerebral ischemia [9]–[11]. Moreover, glutamate (Glu) is an excitotoxic amino acid (EAA) that has been associated with post-ischemia brain damage in animals as well as with progression of ischemic stroke, END and infarct growth in humans [12]–[14].

Two widely used animal models to human brain ischemia are the embolic stroke (ES) and the permanent occlusion of the middle cerebral artery (pMCAO) models in rats [3],[15]–[17]. However, there are no comparative analyses of the inflammatory and excitotoxic response after brain ischemia between focal ischemia rat models and stroke patients in clinical practice. Increasing the knowledge about the differences and similarities between experimental stroke models and stroke in humans could allows for choosing the model that better resembles human for the translational research.

Our primary objective was to study the plasma level profile of TNF-α, IL-6 and Glu in patients with acute cerebral infarction (CI) and compare them with the levels of TNF-α, IL-6 and Glu in two different models of focal ischemia (ES and pMCAO) in order to determine which model is more similar to human. The secondary objectives were: 1) to analyze the relationship of these biological markers with the severity, volume and outcome of the brain infarction in humans and the two stroke models; and 2) to study whether the three biomarkers are also increased in response to damage in organs other than the central nervous system both in humans and in rats.

## Experimental procedures

### Patients

#### Design

This was a prospective case-control study (3:1) whose inclusion criteria for the stroke group (cases) were: patient of any age, symptoms of non-lacunar brain infarction within the previous 12 hours and a brain CT that ruled out any other cause of the neurological deficit. The brain CT was repeated within the first week to demonstrate the presence of a brain infarction and to measure its volume. The hypodensity volume (mL) was calculated in the second scan according to the formula 0.5*XaXbXc* (where *a* and *b* are the largest perpendicular diameters measured in CT and *c* is the height). Patients with symptoms or signs of an acute infection on admission as well as those with a transient ischemic attack (TIA), lacunar syndrome (pure motor stroke/hemiparesis, ataxic hemiparesis, dysarthria/clumsy had, pure sensory stroke and mixed sensorimotor stroke) or symptoms of brainstem infarction were excluded. Furthermore, patients treated with intravenous thrombolysis or intra-arterial reperfusion therapies were excluded. The controls were patients with acute non-neurological diseases with symptom onset within the previous 12 hours. Cases and controls were matched by gender and age (±5 years). Exclusion criteria for the control group were: history of acute stroke in the last year or current episode of stroke, focal or global neurological symptoms attributable to any central nervous system lesions (whether demonstrated or not) and more than one acute disease at the time of the study. Common exclusion criteria for both groups were: underlying severe conditions including bronchial and heart disease requiring more than 3 hospital admissions in the last year or domiciliary oxygen therapy; disseminated or terminal stage cancer in any location; connective tissue disorders with current activity at the time of evaluation; previous chronic inflammatory diseases such as chronic bronchitis; treatment with anti-inflammatory drugs or calcium channel blockers at symptoms onset; dementia or other deteriorating conditions that preclude an acceptable basal functional status, as determined by Barthel Index (BI) scores lower than 90 and modified Rankin scale (mRS) scores higher than 1; and a history of illicit drug abuse.

#### Patient management

Both cases and controls were selected among those who came to the emergency department, according to the inclusion and exclusion criteria. Stroke patients were evaluated by a neurologist and managed in a Stroke Unit following the acute stroke management guidelines [18] with special attention to the surveillance of blood pressure, serum glucose levels and body temperature. The initial assessment of the neurological deficit by means of the National Institute of Health stroke scale (NIHSS) was performed on admission and every 24 hours during hospitalization by certified neurologists. Control patients were evaluated following a standard protocol of management depending on the clinical symptoms. The score in the APACHE II (Acute Physiology and Chronic Health Evaluation II) scale score was registered in cases and controls to establish disease severity [19].

Demographic data, previous vascular risk factors and previous treatments were recorded for both groups and registered in a specific database. Furthermore, functional outcomes at 3-months were evaluated by means of the mRS and BI [20]. The mRS is a scale defined by seven grades (0 indicates no symptoms; 1: no disability, despite symptoms; 2: slight disability; 3: moderate disability; 4: moderately severe disability; 5 severe disability; and 6 death). The BI consists of 10 items that measure a person’s daily functioning, specifically the activities of daily living and mobility. The items include feeding, moving from wheelchair to bed and return, grooming, transferring to and from a toilet, bathing, walking on level surface, going up and down stairs, dressing, continence of bowels and bladder. The total scores ranges from 0 to 100, where 0 is the highest functional dependence and 100 the maximum independence.

The study was approved by the local Ethical Committee of La Paz University Hospital. All patients, or next kin if patient was unable to consent, signed the informed consent before the inclusion in the study. The informed consent followed the standards of the Spanish law (Boletín Oficial del Estado of November 15, 2002).

#### Blood sample management and biochemistry analysis

Laboratory analyses were performed within the first 12 hours from the onset of symptoms and at 72 hours, both in case and controls. Blood samples were extracted by peripheral venous puncture, collected in glass tubes with EDTA K3, centrifuged at 3000g for 15 minutes and the supernatant frozen at –80°C for storage in the biochemistry department. Plasma levels of Glu and other amino acids were measured for physiological amino acids by High Performance Liquid Chromatography (HPLC) following the Pico-Tag© method (Waters Associates) [21] with minor modifications [22]. The reference normal plasma range for Glu in our laboratory was 25 to 110 μmol/l. The pro-inflammatory cytokines IL-6 and the TNF-α were determined by ELISA (Inmunogenetics, S.A.U.). The biochemistry physician was blind to the patients’ clinical characteristics, including their allocation to case or control groups.

### Animals

The procedure was carried out at the Neuroscience and Cerebrovascular Research Laboratory at a university hospital. Animal handling complied with Spanish and European guidelines (*Boletín Oficial del Estado* of March 18, 1988, and 86/609/EEC and 2003/65/EC European Council Directives). All experiments were performed in compliance with guidelines of our Ethical Committee for the Care and Use of Animals in Research (La Paz University Hospital). The experiments were designed to use the smallest number of animals and to minimize their suffering in accordance with the ethical standards of the Helsinki Declaration of 1975. The results are reporting following the ARRIVE (Animal Research: Reporting *In Vivo* Experiments) guidelines.

#### Subjects

Adult male Long-Evans rats (weight 250 to 300g) were used. Rats were housed with free access to food and water and at a room temperature of 21 ± 2°C, relative humidity of 45 ± 15% and a 12h light/ dark cycle (7:00-19:00).

#### Experimental groups

We conducted a randomized, blind study. Rats were assigned to five groups: 1) ES (n = 10), which underwent a right internal carotid artery (ICA) embolization with autologous clot; 2) sham-operated group (n = 6) without embolization; 3) pMCAO (n = 6) with permanent MCAO together with transient bilateral common carotid artery ligation for 60 min; 4) sham-operated group (n = 6) without pMCAO; and 5) Controls which underwent tissue stress by leg compression (LC) for 180 min (n = 6). These animals were subjected to limb muscle compression in back paw without carotid or cerebral damage.

After 72h, animals were re-anesthetized for euthanasia by transcardial perfusion with a saline solution followed by fixing solution (4% paraformaldehyde and 0.1% glutaraldehyde in 10% buffered formalin phosphate).

#### ES model: surgical procedures

Anesthesia was induced by a solution of ketamine (25 mg/mL), diazepam (2 mg/mL), and atropine (0.1 mg/mL) at a dose of 2.5 ml/kg by intraperitoneal injection. Analgesia was provided by meloxicam 2 mg/kg by a subcutaneous route. The femoral vein and artery were cannulated for continuous monitoring of physiological parameters (glycemia, blood gases, blood pressure and heart rate) (Monitor Schiller AG CH 6340BAAR), and for extraction of samples. Body temperature was also monitored and maintained at 36.5 ± 0.5°C. The external carotid artery (ECA) was also cannulated to introduce embolus. This consisted of a 3 mm long by 0.4 mm wide thrombus obtained from arterial blood coagulated in a polyethylene tube (Centracath Vygon 19 G, inside diameter 0.5 mm) at 37.5°C for 40 min. As previously described [15],[23], the thrombus was introduced into the ICA through a catheter located in the ECA, enabling the blood flow to impel it up to the bifurcation of the intracranial ICA where it impacts due to its diameter, causing interruption of blood flow to the middle cerebral artery (MCA). The location of the clot and proper occlusion of MCA were verified by an angiography. Angiography (Stenoscop General Electric. Exposure: 40 Kv, 0.5 mA) with 0.3 ml of non-ionic contrast (Iohexol Omnitrast 300 Schering) was performed at 20 min after embolization to verify the arterial occlusion and again at 120 min to check whether recanalization had occurred. Animals that did not present arterial occlusion after the first angiography or those that spontaneously recanalized (animals that show MCA patency on the second angiogram) were rejected. Using these criteria, we ensured that all animals included in the study had an MCA occlusion and did not present early reperfusion.

The sham-operated animals underwent the entire surgical procedure except for embolization.

#### pMCAO model: surgical procedures

The anesthesia induction and the monitoring of physiological parameters were similar to those previously described for ES.

The surgical procedure to induce permanent focal cerebral ischemia was a variant of that described by Chen et al [16] and Liu et al [17]. A small craniectomy was made above the rhinal fissure over the right MCA branch, which was permanently ligated just before its bifurcation between the frontal and parietal branches with a 9-0 suture. Complete blood flow interruption was confirmed using an operating microscope. Both common carotid arteries were then transitorily occluded for 180 min. A thermistor probe was placed under the temporal muscle and over the cerebral artery region to measure brain temperature. Transient occlusion of common carotid arteries helps to reduce the variability of infarct volume in this model [24].

The sham-operated animals underwent the entire surgical procedure except for MCA ligation.

#### Physiological monitoring: mortality

In all animals, the femoral artery was cannulated during surgery and ischemia for continuous monitoring of physiological parameters (glycemia, blood gases and blood pressure) (Monitor Omicron ALTEA RGB medical devices). Temperature was maintained at 36.5 ± 0.5°C. A deviation of less than 20% from normal mean values of physiological parameters was accepted; animals with values outside these normal limits were rejected.

In the ES, 4 rats died before the 72^th^ hour due to the severity of the brain infarction, and they were excluded from the analysis. There was no mortality before the sacrifice in the pMCAO and LC rats.

#### Functional evaluation

Before the procedure and at 24 and 48 hours after surgery and leg compression, each animal was given a score on the neurological scale described by Rogers [25],[26]: 0 = No deficit; 1 = failure to extend left forelimb; 2 = decreased grip of the left forelimb while tail pulled; 3 = spontaneous movement in all directions, contralateral circling if pulled by the tail; 4 = circling or walking to the left; 5 = movement only when stimulated; 6 = unresponsive to stimulation; 7 = death.

All animals were weighed preoperatively and immediately before the animals were euthanized at 72 hours. Post-operative weight loss as a percentage of preoperative weight was calculated as: 100 × (preoperative weight – pre-euthanization weight)/preoperative weight.

#### Evaluation of infarct volume by hematoxylin-eosin (H&E)

After euthanization, the brains were removed and fixed in 10% buffered formalin for 24h at 4°C. Brains were sectioned at the optic chiasma and at the infundibular stalk. The resultant blocks of brain between these two cuts were then embedded in paraffin and sectioned into 5 μm-thick coronal slices. Every twentieth slice (for a total of four slices [numbers 1, 21, 41 and 61], which were separated by 100 μm from each other) was stained with H&E. H&E staining allows for the identification of ischemic lesions as well-defined pale areas. The infarct volume was thus measured for these sections as previously described [24],[27]. Lesion volumes were calculated as a percentage of the volume of the contralateral hemisphere using the following formula: % lesion volume = (volume of the contralateral hemisphere – ipsilateral intact volume)/volume of contralateral hemisphere × 100.

#### Cell death

Cell death was assessed by marking nuclear DNA fragments *in situ* by immunohistochemistry using the TUNEL method (biotin-dUTP nick end-labeling mediated by terminal deoxynucleotidyl transferase; TdT-FragEL DNA fragmentation detection kit, Oncogene Research Products), following the manufacturer’s instructions, and counterstaining with methyl green. TUNEL-positive cells were counted in all animals in the same predetermined slice of brain located in the central infarct area (slice number 46) using a 40X objective on an optic microscope (Olympus) with analysis software (Image-Pro Plus).

In the ES, the TUNEL-positive cells were counted in the frontal, lateral and piriform areas of the cortex, and in the medial and lateral striatum. The mean of the five counts in each area was calculated. Counts were made both in the embolized and contralateral hemispheres. The results were presented as the total for the embolized hemisphere and then separately for the cortex and striatum [23].

In the pMCAO, we identified cells death in the cortex of both hemispheres based on their nuclear morphology and the dark color [26].

#### Quantification of biochemical markers

Blood samples were obtained at 3 and 72 hours after ischemia, collected in plastic tubes containing EDTA and then immediately centrifuged. Plasma was frozen at -80°C until analysis.

Glu was determined by HPLC as previously described for humans. The pro-inflammatory cytokines, IL-6 and TNF-α, were determined by ELISA (Inmunogenetics, S.A.U.).

#### Data analysis

Statistical analyses were performed with the SPSS package 15.0 for Windows (SPSS Inc., Chicago, Illinois, USA). A univariate analysis was performed with the *X*^2^ test for dichotomous variables. Continuous variables were tested using the *t*-test, the Mann-Whitney or Wilcoxon test when appropriate. The Mann-Whitney, and Kruskal-Wallis tests were used to compare the values of physiological parameters, functional evaluation scores, lesion volumes, number of TUNEL positive cells and plasma levels for Glu and inflammatory cytokines between the study groups, and Wilcoxon test for comparison within study groups. The Spearman correlation coefficient was used to analyze the relationship between Glu and cytokines, infarct size, number of TUNEL positive cells and functional evaluation. In patients, the correlations were adjusted by the brain infarct size, dividing the sample into two groups: large infarcts (third tertile) and medium sized infarcts (first and second tertile). *P*-values less than 0.05 were considered significant.

## Results

### Patients

Fifty-one acute non-lacunar stroke patients (cases) and seventeen acute non-neurological diseases patients (controls) were included in the study. Baseline characteristics, vascular risk factors, disease severity and 90-day outcomes are shown in Table 1. Cases and controls had the same gender distribution (47% males) and similar age 73 ± 10.3 years vs. 70.4 ± 15 years, *P* = 0.353). Furthermore, there were no differences in the blood extraction times and vascular risk factors, except for atrial fibrillation, which was more common in the cases than in controls (43.1% vs. 0%, *P* = 0.001). APACHE II scores on admission as well as 90-day mRS and BI scores were similar in both groups. Seven patients died in the cases group and zero in controls, although this difference was not statistically significant. The median (IQR) brain infarction size in the cases group was 27.8 (78) ml.

**Table 1:** Baseline characteristics, vascular risk factors, stroke etiology, stroke severity, in-hospital complications and 90-day outcomes in humans

	**Stroke**	**Controls**	***P***
	**(n = 51)**	**(n = 17)**	
*Baseline, demographic data and risk factors*			
Men, n (%)	24 (47)	8 (47)	1
Age, mean (SD)	73.5 (10.3)	70.4 (15)	0.353
Symptoms onset to first blood sample, h; mean (SD)	4.8 (3.3)	5 (3.3)	0.795
Symptoms Onset to second blood sample, h; mean (SD)	71.2 (4.9)	73.5 (4.1)	0.394
Systolic blood pressure on admission, mean (SD)	154.3 (19)	149.2 (32.3)	0.467
Blood glucose, mmol/L; median (IQR)	123 (38)	133 (106)	0.100
Hypertension, n (%)	34 (66.7)	12 (70.6)	0.765
Diabetes Mellitus, n (%)	10 (19.6)	6 (35.3)	0.187
Hyperlipidemia, n (%)	17 (33.3)	7 (41.2)	0.558
Atrial Fibrillation, n (%)	22 (43.1)	0 (0)	0.001
Current smoking, n (%)	13 (25.5)	5 (29.4)	0.751
Alcohol abuse, n (%)	2 (3.9)	2 (11.8)	1
Coronary heart disease, n (%)	8 (15.7)	2 (11.8)	1
Peripheral arterial disease, n (%)	1 (2)	2 (5.9)	0.440
*Disease severity*			
NIHSS score on admission, mean (SD)	11.3 (6)	-	-
APACHE II score on admission, mean (SD)	7.4 (2.6)	7 (2.5)	0.555
*90-days outcome*			
mRS 0-2, n (%)	27 (53)	10 (58.8)	0.723
Barthel index ≥ 90, n (%)	22 (43.1)	6 (35.3)	0.569
Mortality, n (%)	7 (13.7)	0 (0)	0.175
*Brain infarction size, ml; median (IQR)*	27.8 (78)	-	
*First and second tertile, ml, range*	3 - 40	-	
*Third tertile, ml, range*	50 - 405	-	

NIHSS indicates National Institutes of Health Stroke Scale; IQR, interquartile range; mRS, modified Rankin Scale.

The etiological stroke subtypes in the cases group were: atherothrombotic (41.2%), cardioembolic (45%), undetermined cause (11.8%) and unusual origin (1.9%). The diseases in the control group were: bone fracture (52.9%), acute myocardial infarction (41.1%) and pulmonary embolism (5.8%).

#### Biochemical markers

**Figure 1:**
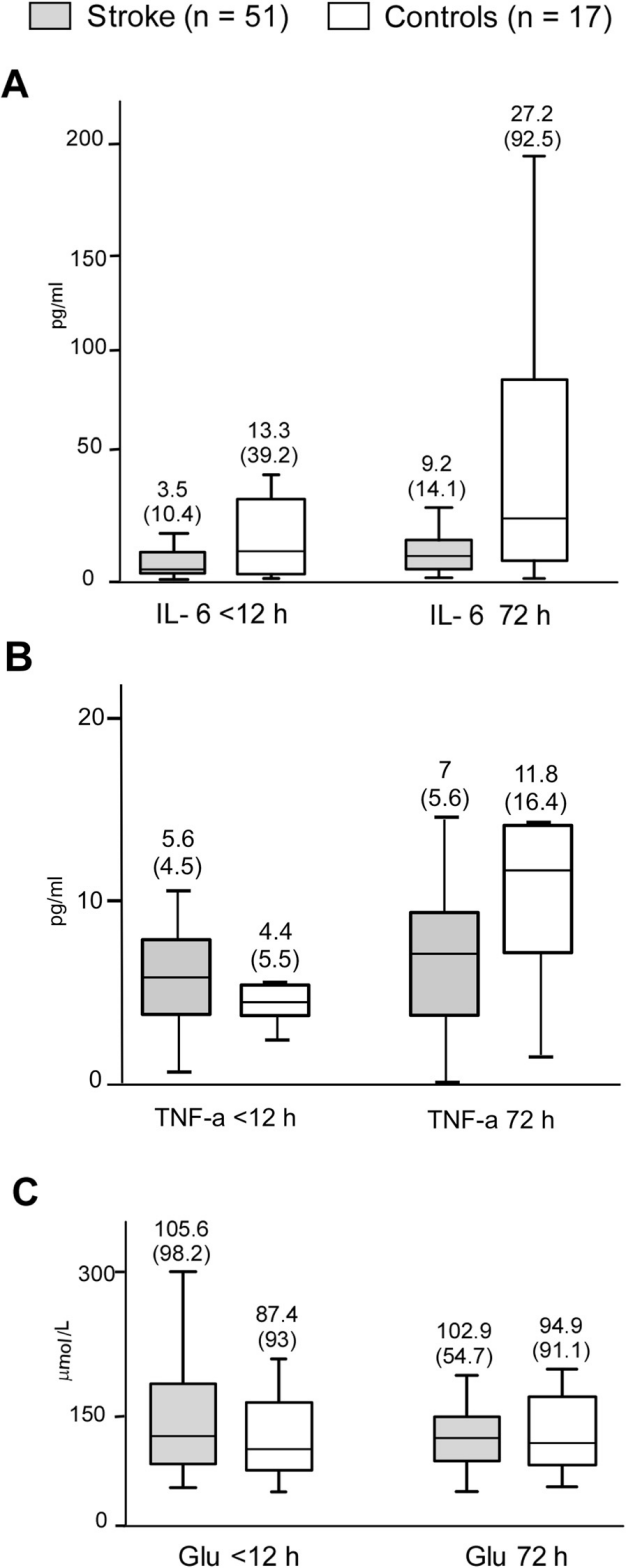
**Plasma biochemical markers in patients, median (IQR). A**: IL-6 plasma levels. **B**: TNF-a plasma levels. **C**: Glu plasma levels. Normal values range: IL-6 ≈ 1-6 pg/ml [28],[29], TNF-a ≈ 5-10 pg/ml [28],[30], Glu ≈ 20-60 μmol/L [31],[32]. IQR means interquartile range; IL-6, interleukin 6; TNF-a, TNF-alpha; Glu, glutamate plasma levels.

Figure 1 shows the biochemical markers in patients. The cases group tended to present lower IL-6 plasma levels than controls in the first measurement (*P* = 0.068), and significantly lower levels in the second measurement (*P* = 0.038). Furthermore, IL-6 plasma levels rose over time in both groups but were significantly only in the cases (*P* = 0.007) (Figure 1A). TNF-α plasma levels were similar in the cases and control groups in the <12 h and the 72 h measurement. Moreover, TNF-α plasma levels increased over time in the cases and control groups, although did not reached the statistical significance (Figure 1B). Glu plasma levels were similar in the cases and control groups in both the first and the second measurement. Furthermore, the Glu levels tended to decrease in the cases group (*P* = 0.053) and did not differ over time in the control group (Figure 1C).

Finally, stroke patients were divided according to the timing of the first blood sample: <6 hour (35/51) and 6-12 hours (16/51). When comparing the <6 h and the 6-12 h groups, there were no statistically differences in plasma levels (median [IQR]) of IL-6 (3.3 [11] vs. 4 [9.6]), Glu (118.6 [101] vs. 87 [72]) and TNF-α (5.3 [4] vs. 6 [10.4]) (P NS).

#### Correlations between biochemical markers, stroke severity, volume of infarct and stroke outcome

Table 2 shows the correlations between biochemical markers and clinical and neuroimaging parameters in patients. In stroke patients, correlations were found between IL-6 at <12 h and clinical severity in the case of patients with large infarcts. IL-6 at <12 h showed a tendency towards a positive correlation with 3-month mRS in patients with large infarcts. IL-6 at 72 h correlated with clinical severity, stroke severity, volume of infarct and 3-month mRS in all stroke patients, as well as with stroke severity in patients with medium-sized infarcts. TNF-α levels at <12 h correlated with infarct volume in all stroke patients and in the large subtype. Furthermore TNF-α levels at <12 h correlated with 3-month mRS. Glu levels at <12 h did not show any significant correlation with clinical/neuroimaging parameters in stroke patients. Glu at 72 h tended towards a positive correlation with stroke severity in all patients and showed a significant correlation with stroke severity in patients with medium-sized infarcts.

**Table 2:** Stroke patients: correlations between biochemical markers, stroke severity, volume of infarct and stroke outcome

**Values of spearman rho correlations:**		**IL-6**		**TNF-α**		**Glutamate**	
		**IL-6**	**IL-6**	**TNF-α**	**TNF-α**	**Glu**	**Glu**
		**<12 h**	**72 h**	**<12 h**	**72 h**	**<12 h**	**72 h**
Clinical severity (APACHE II)	All patients	0.261	0.326**	0,130	0,155	−0.076	−0.029
		−0.061	0.484**	−0,085	0,068	0.421*	−0.193
	Large infarcts	0.630***	0.433	0.244	0.347	−0.052	0.149
		−0.369	0.197	−0,821	−0,462	−0.350	−0.727**
	Medium infarcts	−0.066	0.241	0.190	0.044	−0.042	−0.007
		0.261	0.759**	-	0.400	0.374	0.410
Stroke severity (NIHSS)	All patients	0.068	0.497***	0.312	0.247	0.193	0.261*
		-	-	−0.488	−0.488	-	-
	Large infarcts	0.247	0.020	0.388	0.433	0.165	−0.013
		-	-	−0.866	−0.866	-	-
	Medium infarcts	−0.223	0.505***	−0.023	−0.108	0.217	0.371**
		-	-	−0.866	−0.866	-	-
Volume of infarct^§^	All patients	0.207	0.290**	0.476**	0.275	0.120	0.151
	Large infarcts	0.398	0.299	0.592*	0364	0.325	0.324
	Medium infarcts	0.069	−0.113	0.097	0.178	0.001	0.001
Clinical outcome: 3-months mRS	All patients	0.230	0.444***	0.352*	0.150	0.101	0.006
		**-**	-	0.546	−0.095	-	-
	Large infarcts	0.453*	0.455	0.370	0.283	0.235	0.295
		-	-	0.105	0.316	-	-
	Medium infarcts	0.042	0.273	0.338	0.120	−0.023	−0.080
		**-**	-	0.316	−0.738	-	-
Clinical outcome: 3-months Barthel index	All patients	−0.121	−0.245	−0.152	−0.109	−0.164	−0.017
		−0.492*	−0.303	−0.536	−0.009	0.092	0.433*
	Large infarcts	−0.366	−0.366	0.119	−0.444	−0.268	0.000
		−0.449	−0.338	−0.200	−0.300	−0.570	0.591*
	Medium infarcts	−0.053	−0.078	−0.262	0.168	−0.042	0.006
		−0.702*	0.025	−0.316	0.738	0.368	−0.495

The numbers in the upper line of each row correspond to stroke patients and the line below to controls.

*P < 0.1; **P < 0.05; ***P < 0.01. Results with *P *< 0.1 are in bold

^§^Only stroke patients

APACHE II, Acute Physiology and Chronic Health Evaluation II scale; NIHSS, National Institutes of Health Stroke Scale.

In the control group, IL-6 at <12 h tended towards a negative correlation with the 3-month BI score. IL-6 at 72 h also correlated with clinical severity in the control group. TNF-α did not correlate with any of these parameters in the control group. Glu at <12 h tended towards a correlation with clinical severity in all control subjects, while Glu at 72 h correlated negatively with clinical severity in large infarct control group. Moreover, Glu at 72 h correlated positively with clinical outcome measured by the BI (Table 2).

### Animal models

Physiological parameters remained within normal limits throughout the procedure and there were no significant differences between the groups.

**Figure 2:**
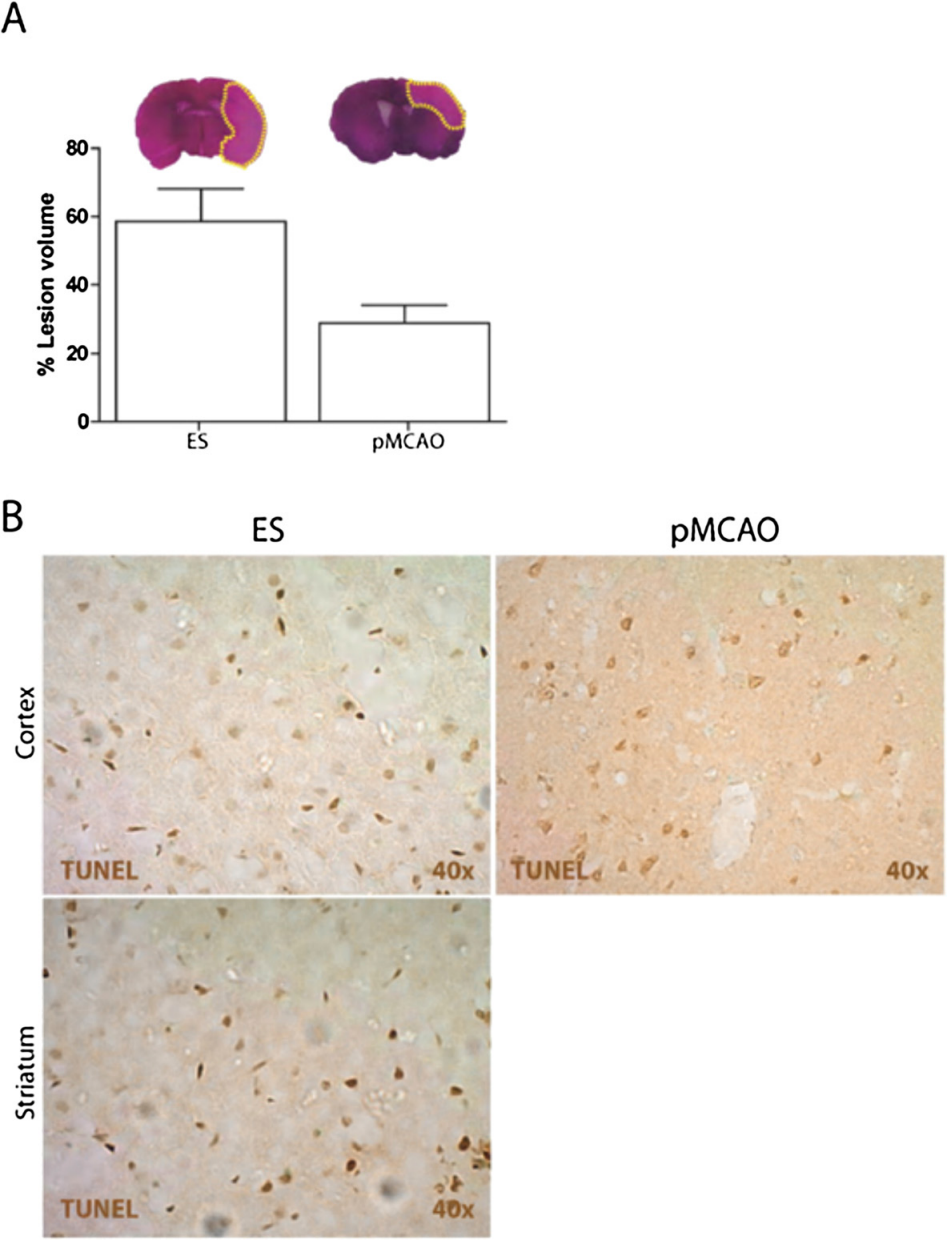
**Infarct volume and cell death. A)** Infarct volume: H&E staining allows the identification of ischemic lesions as well-defined pale areas for both models (ES and pMCAO). Infarct volume was significantly higher in the ES than in the pMCAO model (bar graph). **B)** Cell death (Tunel positive cells): In the ES model, the quantification of the cell death is presented separately for the cortex and striatum whereas in the pMCAO model only the cortex is presented.

Six rats underwent the ES, six the pMCAO and another six the LC. The infarct volume, cell death, functional evaluation and post-operative weight loss in ES and pMCAO are shown in Table 3. ES showed brain infarct affecting the cortex and the striatum, but the infarcts in the pMCAO affected only the cortex. Thus, the infarct volume was significantly higher in the ES (infarct volume median in terms of percentage of right hemisphere) (Table 3 and Figure 2A). However, the percentage of post-operative weight loss was similar in both groups. Cell death measured as the number of TUNEL-positive cells in the infarction was higher in the cortex of pMCAO rats (Table 3 and Figure 2B). Furthermore, the functional evaluation was poorer in the ES at 24 h, 48 h and 72 h (Table 3).

**Table 3:** Infarct volume, cell death, functional evaluation and post-operative weight loss in the embolic stroke (ES) and the permanent middle cerebral artery occlusion (pMCAO) models

	**ES (*n*** **= 6)**	**pMCAO (*n*** **= 6)**	***P***
***Volume of infarct in percent of the contralateral hemisphere, median (IQR)***	58.6 (9.6)	29 (5.1)	<0.001
***Cell death as number of TUNEL positive cells***			
Cortex, median (IQR)	103 (20.1)	133.3 (18.1)	0.022
Striatum, median (IQR)*	59.1 (10.4)	-	-
Functional evaluation**			
At 24h, median (IQR)	4 (1)	3 (0)	0.009
At 48 h, median (IQR)	4 (1)	3 (0)	0.009
At 72 h, median (IQR)	4 (1)	3 (1)	0.041
*Percent post-operative weight loss, mean (SD)*	34.8 (11.5)	42.8 (12)	0.286

*Striatal infarction is only present in the ES model

**Rogers scale score.

#### Biochemical markers

**Figure 3:**
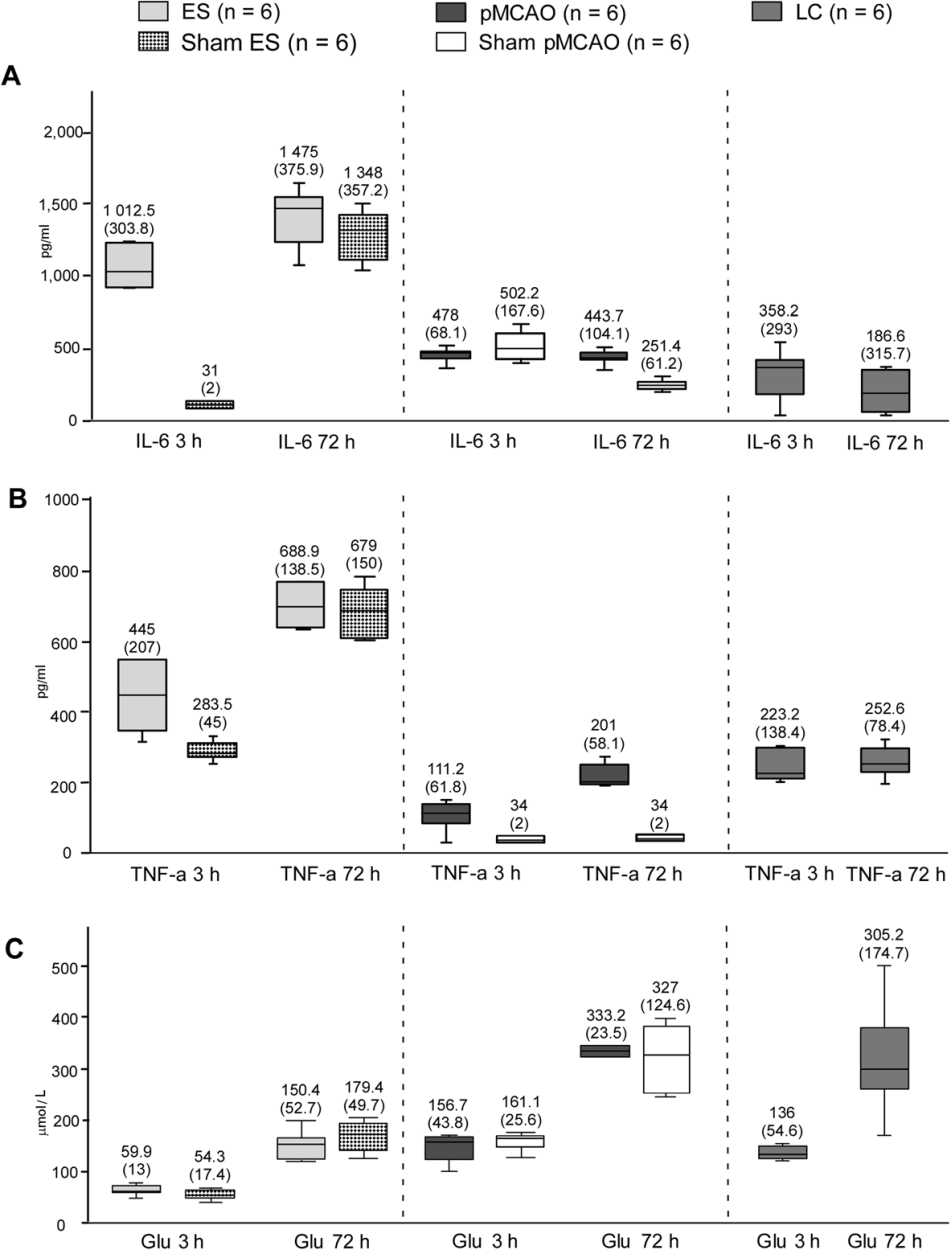
**Plasma biochemical markers in animal models, median (IQR). A**: IL-6 plasma levels. **B:** TNF-α plasma levels. **C:** Glu plasma levels. IQR means interquartile range; ES, embolic stroke model; pMCAO, permanent middle cerebral artery occlusion model; LC, local compression model; IL-6, interleukin 6; TNF-a, TNF-alpha; Glu, glutamate.

The biochemical marker levels in the five groups of rats are shown in Figure 3. The ES had higher IL-6 plasma levels than the pMCAO rats in both the 3 h and 72 h measurements (*P* = 0.002). Furthermore, the IL-6 plasma levels were higher in the ES than the LC group (*P* = 0.002). The IL-6 plasma levels were higher in the ES than in its sham at 3 h (P = 0.002) but not at 72 h. There were no differences in any IL-6 plasma level between the pMCAO and its sham. IL-6 levels at 3 h were similar between pMCAO and LC but, at 72 h, were higher in pMCAO (*P* = 0.009). The IL-6 plasma levels increased significantly over time in the ES (*P* = 0.046), but not in the pMCAO or LC groups (Figure 3A).

Regarding the TNF-α (Figure 3B), the ES showed higher plasma levels than the pMCAO at 3 h (*P* = 0.002) and 72 h (*P* = 0.002). Moreover, TNF-α levels at 3 h and 72 h were higher in ES than in LC (*P* = 0.026 and *P* = 0.002, respectively) rats. TNF-α levels were lower in pMCAO than in LC at 3 h (*P* = 0.002) but not at 72 h. The TNF-α plasma levels were higher for the ES than for its sham at 3 h (*P* = 0.004) but not at 72 h, and higher for the pMCAO than for its sham both at 3 h (*P* = 0.015) and at 72 h (*P* = 0.002). Furthermore, TNF-α increased over time in both ischemic models (*P* = 0.027 for the ES, and *P* = 0.028 for the pMCAO) but remained stable in the LC group.

Regarding the Glu (Figure 3C), the ES presented lower plasma levels than the pMCAO at 3 h (*P* = 0.002) and at 72 h (*P* = 0.002). There were no differences in Glu levels between the ES or pMCAO and their corresponding shams. Moreover, Glu levels were lower in ES than in LC at 3 h (*P* = 0.002) and 72 h (*P* = 0.004), although there were no significant differences between pMCAO and LC. The plasma Glu levels increased over time in ES (*P* = 0.028), pMCAO and LC (*P* = 0.028).

#### Correlations between biochemical markers, functional evaluation, volume of infarct and cell death

In the ES, significant correlations were found between IL-6 levels at 3 h and cell death in the cortex. Furthermore, there was a tendency towards a correlation between Glu at 3 h and cell death in the cortex. TNF-α levels at 3 h tended to be correlated with cell death in the cortex. Moreover, Glu levels at 72 h were positively correlated with infarct volume (Table 4).

**Table 4:** Models of stroke: correlations between biochemical markers, functional evaluation, volume of infarct and cell death

**Correlations: values of spearman R**	**IL-6**		**TNF-α**		**Glutamate**	
	**IL-6 3h**	**IL-6 72h**	**TNF-α 3h**	**TNF-α 72h**	**Glu 3h**	**Glu 72 h**
**Clinical score pre-euthanasia (72 h)**	0.171	−0.600	0.348	−0.522	0.429	0.388
	0.507	0.169	0.676	0.338	−0.169	0.169
**Volume of infarct as percentage of contralateral hemisphere**	0.348	0.319	−0.088	−0.794	0.116	0.943***
	0.058	−0.087	0.522	0.000	−0.232	0.232
**Cell death TUNEL in cortex**	0.928***	−0.058	0.765*	−0.353	0.783*	0.486
**TUNEL in striatum**^**†**^	−0.714	−0.257	−0.714	−0.886**	0.086	−0.257
	−0.580	0.290	−0.294	−0.177	−0.348	0.429
	-	-	-	-	-	-

The upper line of each row corresponds to the embolic stroke (ES) model and the line below to the permanent middle cerebral artery occlusion (pMCAO) model. *P < 0.1; **P < 0.05; ***P < 0.01. Results with *P*< 0.1 are in bold

^**†**^The pMCAO model presents infarctions affecting the cortex but not the striatum.

On the other hand, the only significant correlation found in the pMCAO was a negative correlation between TNF-α levels at 72h and cell death in the cortex (Table 4).

## Discussion

This study shows significant differences in the biomarker profile between the ES and pMCAO model and, in turn, between them and stroke patients. Of the three biomarkers studied, IL-6 emerges as the most closely related to clinical severity, stroke severity, infarct volume and clinical outcome in stroke patients, although Glu also correlated with stroke severity (but not with outcome) and TNF-alpha levels with infarct volume and outcome. Regarding the two animal models of brain ischemia, the ES showed a higher infarct, IL-6 temporal profile more similar to humans, as well as correlations between the three biomarkers, cell death and infarct volume that were more similar to humans than pMCAO. However, the three biomarkers were nonspecific of brain ischemia both in humans and rats.

It is well known that IL-6 is associated with END [6], greater infarct volumes [7],[33] and poor outcomes [8],[34] in stroke patients. In our results, IL-6 levels increased over time in stroke patients and were correlated with clinical severity, stroke severity, infarct volume and long-term clinical outcomes. Even in medium-sized brain infarction, IL-6 levels correlated with stroke severity, confirming the predictor value of this cytokine in ischemic stroke patients. However, the elevation of IL-6 levels was also correlated with increased clinical severity and poorer outcomes in control patients, showing that this biomarker is not specific to cerebral ischemia. Indeed, IL-6 is a multifunctional cytokine produced by various types of cells and regulates the immune response, hematopoiesis, the acute phase response and inflammation [35] and is related to mortality and severity of acute diseases in the Emergency Department [36]. In the present study, IL-6 levels were correlated with cell death in cortex in the ES but not in the pMCAO. Furthermore, IL-6 increased with time in humans and ES, but not in pMCAO. Interestingly, IL-6 levels were markedly higher in ES than in pMCAO and LC, which may be a marker of a more aggressive surgical procedure in ES and may also explain the higher mortality and larger stroke sizes in this model.

TNF-α, another classic pro-inflammatory cytokine, is released early into cerebrospinal fluid and blood in the acutely infracted brain of humans [10] and rodents [37]. In this study, TNF-α plasma levels correlated with apoptotic cell death in the cortex of ES rats. Previous studies have shown that TNF-α, together with IL-1β, induces a secondary inflammatory response mediated by IL-6 and IL-8, which appears to exacerbate cerebral ischemic injury [9],[10]. Moreover, TNF-α plasma levels correlated with infarct volume and with stroke outcome. Although there are no previous studies in humans to compare these data, it is known that TNF-α expression is upregulated in response to ischemia and injury [11],[38]. Furthermore, TNF-α plasma levels tended to increase over time and were no different in the cases and controls groups. In our animal models of brain ischemia, TNF-α increased over time in both ES and pMCAO, although they were higher in ES. The increase over time of TNF-α is striking because experimental studies show that the expression of TNF-α is detected as early as 1 hour after focal ischemia [37], peaks within 12 hours and rapidly decreases over the next 12 to 48 hours. However, these studies directly analyzed TNF-α expression in cortex sections whereas the temporal profile of this biomarker in plasma is unknown. In this study, the human controls, the ES shams and the LC model also had temporal increases in TNF-α plasma levels, showing the non-specific nature of this pro-inflammatory cytokine. Indeed, TNF-α serum levels can be increased with time after injury in other tissues, as is the case with severe burns, in both human and rats [35],[39].

In our cohort of stroke patients, Glu plasma levels were correlated with stroke severity. Previous studies have also shown that plasma Glu concentration are associated with stroke severity [13], as well as with early neurological worsening [12], infarct growth and volume of tissue at risk of infarction [14]. Regarding the animal models in this study, plasma Glu levels were correlated with infarct volume and cell death in the cortex, but only in ES, showing a greater similarity to humans. Glu plasma levels tended to decrease with time in stroke patients, which is in accordance with previous studies that show lower levels of this EAA in stable strokes [13],[14]. We did not specifically evaluate, in our patients, the stability of brain infarcts in the first 24 hours; however, initial Glu plasma levels were relatively low (median 105.6 μmol/l). Considering that Glu concentrations >200 μmol/l within the first 24 hours from stroke onset have been associated with early infarct progression and END [13],[14], we expected that the most of the patients had stable brain infarcts. However, in animal models of brain ischemia, Glu levels significantly increase over time, which could be explained by various hypotheses. First of all, the size of the brain infarction may play an important role in Glu release. In our study, the brain infarction in humans was relatively small, with a median volume around 28 ml, which is equivalent to 5% of the volume of the hemisphere [40]. However, the ES model produced large infarctions affecting, on average, more than 50% of the hemisphere. In humans, infarcts consistently greater than 39% of the ipsilateral hemisphere are malignant infarctions and develop substantial edema and progressive infarct expansion [41]. Thus, the elevation of Glu plasma levels over time in ES may indicate, as previously described in humans [14],[42], a progression of the brain infarction, although we have not specifically measured this. Secondly, the pMCAO model produced significantly smaller infarctions (29% of the hemisphere, on average) but showed even greater Glu plasma levels than ES, which also increased over time. This could be explained by the surgical procedure of this model that involves the direct manipulation of the brain. In fact, it has been previously reported that brain stimulation increases EAA levels, both in humans and animal models [43]. On the other hand, Glu levels were similar between stroke patients and controls, as well as between rats with brain ischemia and their corresponding shams, indicating that Glu levels are not specific to brain tissue lesions, which is similar to inflammatory cytokines. Futhermore, Glu levels increased over time in the LC model, even though were above the levels in the ES model. Previous studies have reported that Glu is released after stressful systemic stimulus both in animals [44] and humans [45].

In this study we studied the plasma levels of three biological markers involved in cerebral ischemia without considering recanalization, to avoid the effect of ischemia/reperfusion injury on inflammatory biomarker release. Experimental and clinical studies have shown that the reperfusion may exacerbate the injury initially caused by ischemia by increasing the leukocyte infiltration, activating platelets and complement, developing post-ischemic hyperperfusion and breakdown of the blood-brain barrier [46]. Furthermore, the ischemia/reperfusion injury amplifies the inflammatory response by inducing the release of several proinflammatory cytokines, including IL-6 and TNF-α. To analyze the biomarkers related only to brain ischemia, we included preclinical models of stroke, where the absence of recanalization can be easily monitored, and we excluded stroke patients treated with reperfusion therapies.

This study has some limitations, one of which is the small sample size of humans and rats. Furthermore, the number of controls in the human group is unbalanced relative to the number of strokes. This is the first comparative study of stroke patients and two animal models of brain ischemia, so it was initially planned as a pilot study with the minimum required sample. However, we have found significant similarities and differences between the animal models and humans, as well as the correlations between plasma biomarkers and clinical data. This new information allows us to expand current knowledge about animal models of focal ischemia in order to improve their applications when testing therapies for stroke. Another limitation is the use of similar temporal windows for plasma analyses in both humans and rats, two animals with very different life expectancy. Is it not known how this can affect the comparison of the data. Finally, the methodology used to assess the infarct volume in stroke patients has been developed in the setting of intracranial hemorrhage and its utility in brain infarct is not clear.

In conclusion, the three biomarkers studied in humans (IL-6, Glu and TNF-α) have different temporal profiles and are non-specific to cerebral injury. However, they establish good correlations with disease severity and outcomes. Similarly, IL-6, Glu and TNF-α are non-specific to cerebral ischemia in biomarker studies in rats. The ES model shows higher infarct volumes and inflammatory biomarkers levels than pMCAO, although the latter had increased levels of serum Glu. However ES shows correlations between the biomarkers, cell death and infarct volume more similar to humans than pMCAO. Both models, ES and pMCAO, show differences that should be considered when conducting translational studies. Larger studies are needed to confirm our data and to clarify how the differences found between the two experimental rat models of brain ischemia may affect the success of drug trials.

## Competing interests

The authors declare that they have no competing interests.

## Authors' contributions

PMS drafted/revised the manuscript, including medical writing for content; participated in the study concept or design, analysis or interpretation of data; MGF carried out the experimental studies and drafted the manuscript; BF drafted/revised the manuscript, including medical writing for content; participated in the study concept or design, analysis or interpretation of data; JM drafted/revised the manuscript, including medical writing for content; MALC drafted/revised the manuscript, including medical writing for content; participated in the study concept or design, analysis or interpretation of data; MENL drafted/revised the manuscript, including medical writing for content and EDT drafted/revised the manuscript, including medical writing for content; participated in the study concept or design, analysis or interpretation of data. All authors read and approved the final manuscript.
